# Correction to: Impact of alternative definitions of contemporary groups on genetic evaluations of traits recorded at lambing

**DOI:** 10.1093/jas/skaf062

**Published:** 2025-03-14

This is a correction to: N. McHugh *et al.*, Impact of alternative definitions of contemporary groups on genetic evaluations of traits recorded at lambing, *Journal of Animal Science*, Volume 95, Issue 5, May 2017, Pages 1926–1938, https://doi.org/10.2527/jas.2016.1344

The authors regret that in the published article, there was an error in the reporting of the lambing difficulty score scale. In the text the lambing difficulty score reads:“1 = no lambing assistance/unobserved, 2 = slight assistance, 3 = severe assistance and 4 = veterinary assistance (including caesarean)”.

The correct lambing difficulty score throughout the text should read: “1 = no lambing assistance/unobserved, 2 = voluntary assistance, 3 = slight assistance, and 4 = severe assistance (including veterinary)”.

The first line of the results section, which quotes the percentage per score should read: “26% required voluntary assistance, 8% required slight assistance, and 3% required severe (including veterinary) assistance at lambing”.

The authors confirm that this was an unintended error.

The emendations are outlined in this notice only to preserve the version of record.

